# Anti-Ri Paraneoplastic Neurological Syndrome Presenting with Ocular Flutter in a Patient with Breast Cancer

**DOI:** 10.3390/brainsci15060628

**Published:** 2025-06-11

**Authors:** Francesca Cascone, Federica Stella, Christian Barbato, Antonio Minni, Giuseppe Attanasio

**Affiliations:** 1 Department of Sense Organs, Sapienza University of Rome, Policlinico Umberto I, 00161 Roma, Italyantonio.minni@uniroma1.it (A.M.); giuseppe.attanasio@uniroma1.it (G.A.); 2 Institute of Biochemistry and Cell Biology (IBBC-CNR), Sapienza University Rome, Policlinico Umberto I, 00161 Roma, Italy; 3 Division of Otolaryngology-Head and Neck Surgery, Ospedale San Camillo de Lellis, ASL Rieti-Sapienza University, 02100 Rieti, Italy

**Keywords:** ocular flutter, anti-Ri syndrome, otorhinolaryngology, paraneoplastic neurological syndrome

## Abstract

Ocular flutter is an uncommon ophthalmic finding that may indicate paraneoplastic phenomena, and it is clinically characterized by intermittent bursts of conjugate, horizontal saccades without an intersaccadic interval. Ocular flutter must be differentiated from opsoclonus, which, although also characteristic of certain paraneoplastic syndromes, is instead defined by multidirectional saccades on both the horizontal and vertical planes. This report describes a very rare presentation of anti-Ri syndrome in a patient with an undiagnosed breast cancer, presenting with ocular flutter, dizziness, blurred vision, photophobia, and vomiting. Comprehensive evaluations, including contrast-enhanced brain Magnetic Resonance Imaging (MRI), brain Computed Tomography (CT) scan, ophthalmological assessment, viral serology, complete blood count and thyroid, renal coagulation, hepatic function assessments, vitamin D and B12 levels, were all normal. Upon excluding other potential etiologies for the neurological symptoms, a paraneoplastic origin was considered. Serological tests confirmed the presence of anti-Ri onconeural antibodies, and a whole-body CT scan identified nodules in the right breast. Despite surgical excision of the primary tumor and subsequent medical therapy, there was no improvement in the neurological symptoms. Follow-up evaluations at 2 months, 6 months, 1 year and 2 years revealed persistent vestibular and neurological symptoms, with serum tests remaining positive for anti-Ri antibodies and no clinical or radiological evidence of neoplastic recurrence.

## 1. Introduction

Paraneoplastic neurological syndromes (PNSs) are rare complications of various malignancies, occurring in less than 1% of patients affected by breast cancer, thymoma, ovarian cancer, small cell lung cancer, lymphoma, and testicular cancer [1,2]. These syndromes are immunologically mediated, involving the production of onconeural autoantibodies such as anti-Ri, anti-Yo, anti-Hu, anti-CV2.1, anti-Amphiphysin, and anti-Ma2/Ta, which target antigens within the central nervous system (CNS). Clinical manifestations of PNSs include opsoclonus myoclonus syndrome (OMS), and less typical symptoms such as jaw dystonia, laryngospasms, cranial nerve palsies, and other oculomotor disturbances. Anti-Ri antibodies are particularly associated with subacute neurological paraneoplastic syndromes, which commonly present with opsoclonus-myoclonus syndrome and paraneoplastic cerebellar degeneration with ataxia. The presence of these syndromes should prompt an investigation for an occult malignancy.

Opsoclonus myoclonus syndrome (OMS) is a particularly rare manifestation of cancer, characterized by opsoclonus, myoclonus, ataxia, behavioral, and sleep disorders. Opsoclonus involves rapid, involuntary, multivector (both horizontal and vertical), unpredictable, conjugate eye movements without inter-saccadic intervals. Myoclonus is defined by brief involuntary movements resulting from muscle contractions. In adults, OMS is most commonly associated with small-cell lung cancer (SCLC) and breast cancer [3,4]. Anti-Ri autoantibodies, which target two central nervous system (CNS) antigens, Nova-1 and Nova-2, are the most commonly implicated in this condition. We report a rare case of anti-Ri syndrome associated with breast cancer, presenting with ocular flutter instead of the more common opsoclonus.

Ocular flutter is a disorder of eye movement characterized by involuntary, consecutive saccades in the horizontal plane, occurring without an intervening saccadic interval. In contrast, opsoclonus involves pathological eye movements that occur in both the horizontal and vertical planes [5]. Epidemiological data on this condition are contradictory. As of 1 April 2020, only 40 cases involving at least one movement disorder and documented antineuronal nuclear autoantibody type 2 (ANNA-2/Ri) in serum and/or cerebrospinal fluid had been reported in PubMed [6]. Among these, only 22% were associated with cancer, and merely 11% were linked to breast cancer. There were only two reported cases of an association between breast cancer and ocular flutter [7]. Murphy et al. documented only 56 cases of breast cancer-related paraneoplastic syndromes at their institution (Mayo Clinic, USA) over a period of 20 years, with just 5 cases of anti-Ri-associated breast cancer PNS among 17,725 patients treated during that period [8]. Only one of these cases developed Opsoclonus Myoclonus Syndrome (OMS).

Additionally, Simard et al. published a comprehensive 20-year retrospective case series in 2020, involving 36 patients and a literature review on anti-Ri syndromes in neurology [9]. Most patients (92%) had a cancer diagnosis, with breast cancer being the most prevalent underlying malignancy (61%).

## 2. Case Presentation

**Figure 1:**
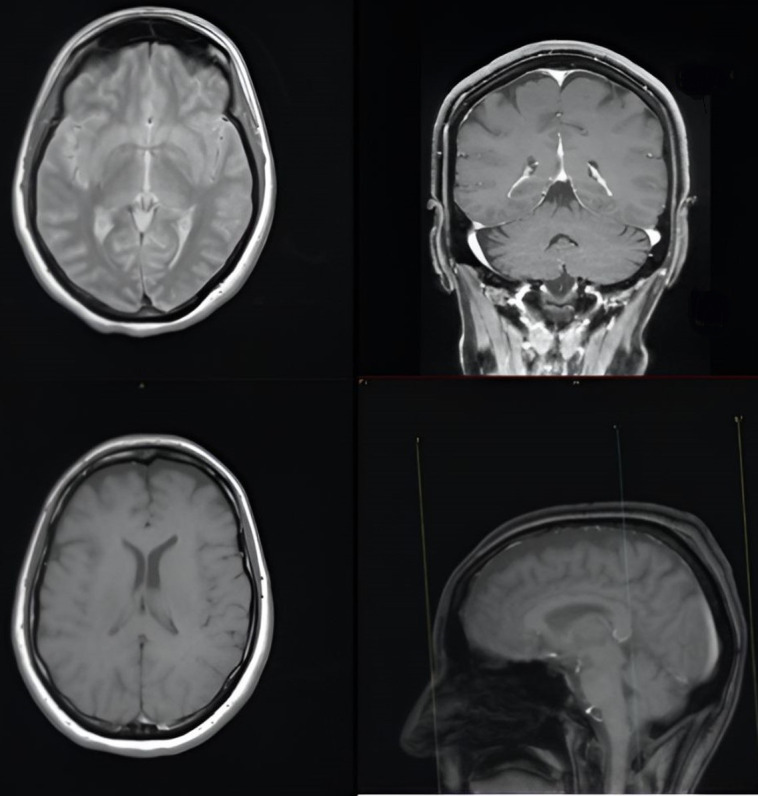
Brain magnetic resonance imaging with contrast.

**Figure 2:**
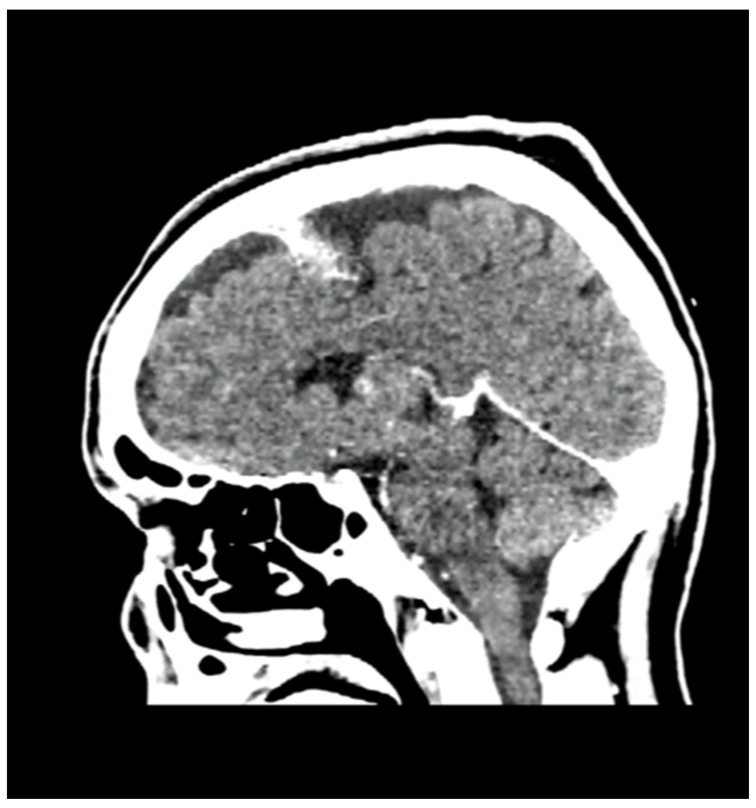
Brain CT scan.

**Figure 3:**
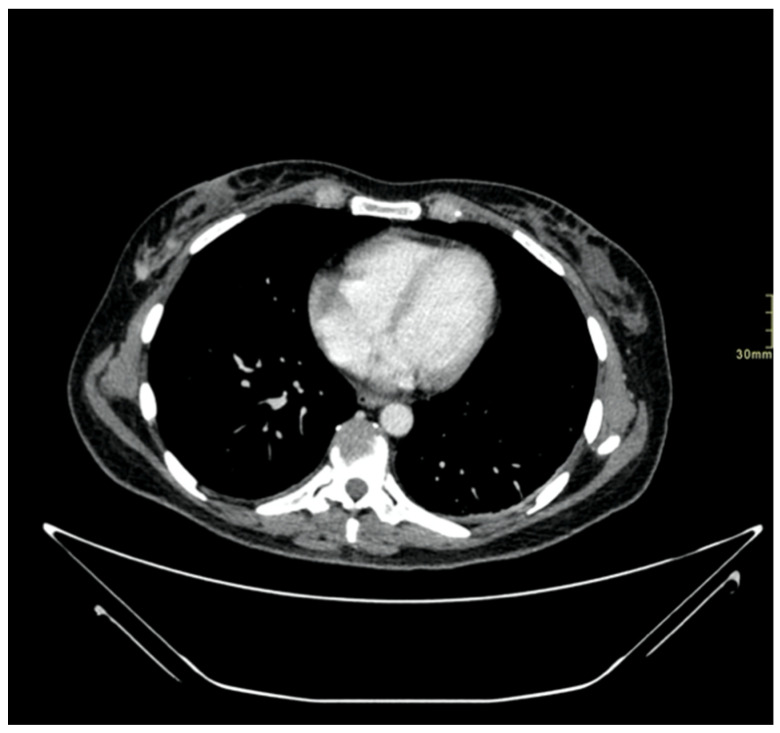
Multiple solid non-calcific nodules (at least 4 in the right QQEE), the largest measuring 13 × 9 mm revealed by the whole-body CT scan.

**Figure 4:**
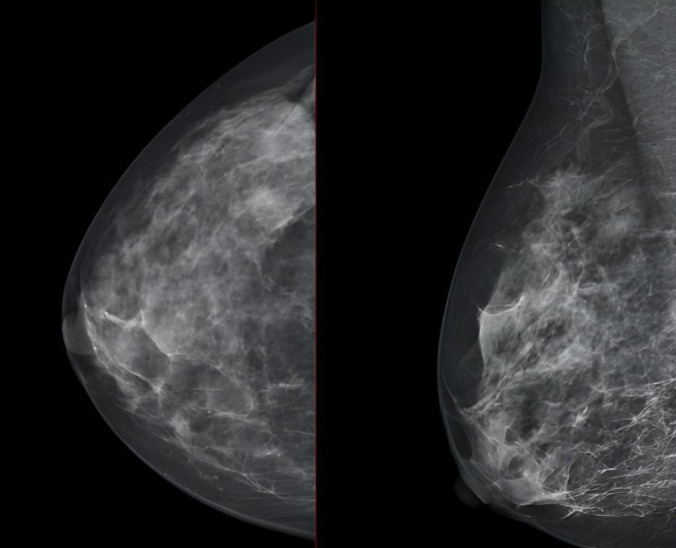
Three nodular opacities with an irregular shape and intranodular microcalcifications at the level of the right QSE showed by mammography.

**Figure 5:**
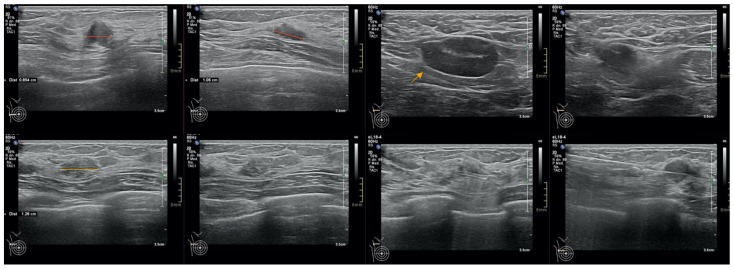
Ultrasonographic image showing irregular, solid, hypoechoic nodules (red lines), suspicious for malignancy. Axillary lymph nodes are also visible (indicated by orange arrows and lines), some of which display altered morphological features.

**Figure 6:**
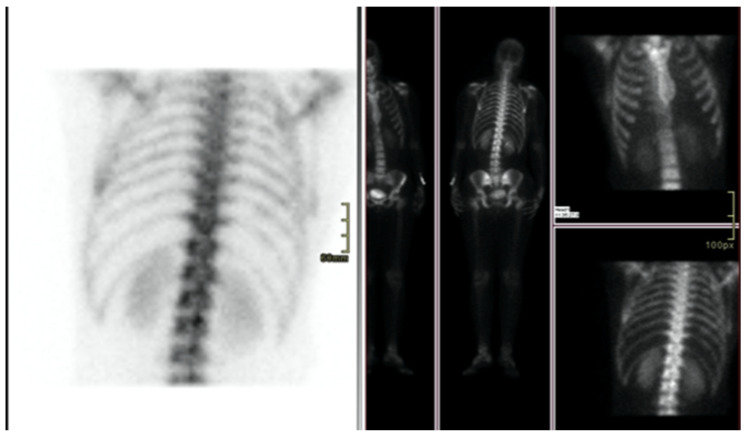
Bone scan not revealed evidence of the disease’s metastatic localization.

**Figure 7:**
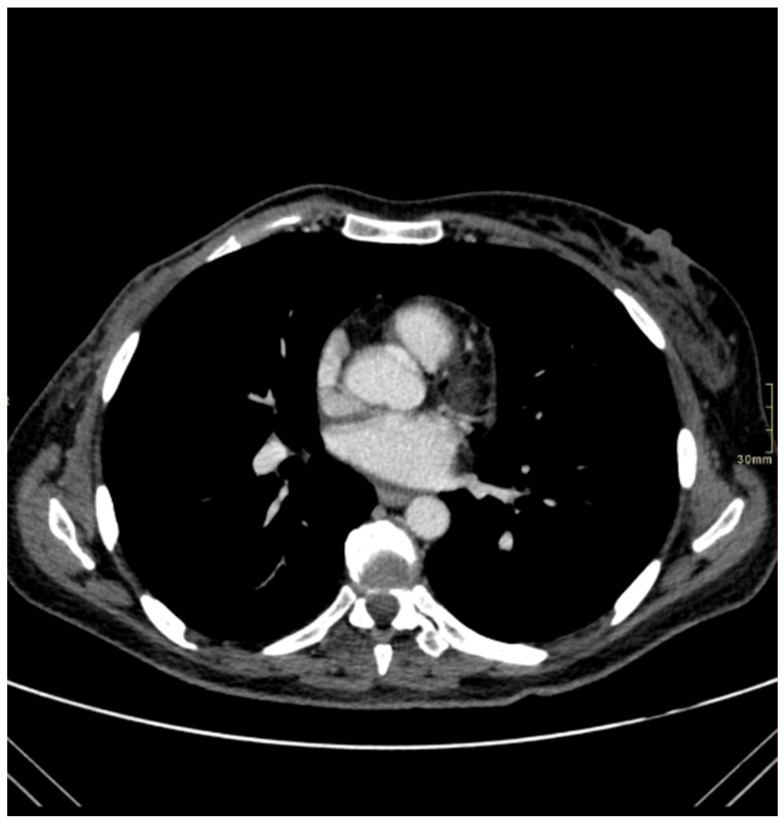
Absence of radiological evidence of neoplastic recurrence in the 2 months follow-up whole-body CT scan.

**Figure 8:**
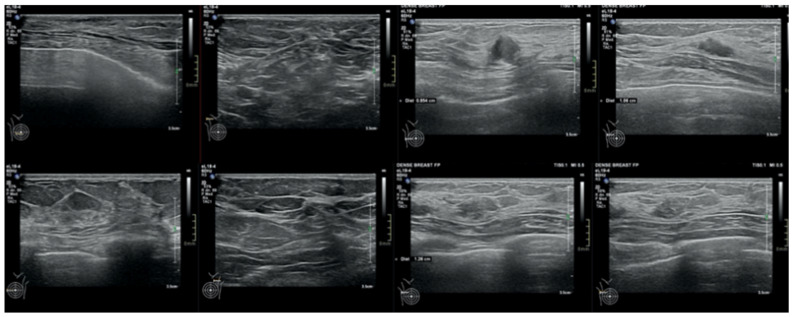
Absence of radiological evidence of neoplastic recurrence in the 2 months follow-up breast ultra-sound.

We report the case of a 47-year-old pre-menopausal Caucasian woman who presented to the Policlinico Umberto I hospital in Rome, with a 6-month history of subjective dizziness, oscillopsia, instability, imbalance, blurred vision, and photophobia, accompanied by severe vomiting, which resulted in severe weight loss (16 kg over 7 months) (Table 1). The medical history was significant for only hypothyroidism and her family history was positive for a sister with breast cancer. Clinical examination of our patient revealed marked fluctuations without prevalence to the side during Romberg and Fukuda tests, both with open and closed eyes. In video-oculoscopy (Supplementary Materials Video S1), the assessment of extraocular motility demonstrated isolated and intermittent episodes of low-amplitude, high-frequency horizontal saccades occurring without an intersaccadic interval, consistent with the clinical presentation of ocular flutter [10]. A comprehensive evaluation was performed, including contrast-enhanced brain Magnetic Resonance Imaging (MRI) (Figure 1), brain Computed Tomography (CT) scan (Figure 2), ophthalmological assessment, viral serology, autoimmune panel, complete blood count, and assessments of thyroid, renal, coagulation, hepatic function, vitamin D and B12 levels; all results were within normal limits. Paraneoplastic syndrome (ENS) sometimes precedes the diagnosis of cancer, necessitating a screening to detect an unidentified tumor. After excluding all other potential causes of the neurological symptomatology, a paraneoplastic origin was considered. Paraneoplastic autoantibody panels (anti-Ri, anti-Yo, anti-Hu, anti-CV2.1, anti-Amphiphysin, and anti-Ma2/Ta) resulted positive for anti-Ri onconeural antibodies with a value of 1:126 (51–256 strong positivity). A whole-body CT scan revealed multiple solid non-calcific nodules (at least 4 in the right breast QQEE), the largest measuring 13 × 9 mm, and right axillary lymphadenopathy measuring 24 mm (Figure 3). Mammography showed at least three nodular opacities with an irregular shape in the external superior quadrant of the right breast. The biggest ones were 13 mm (with intranodular microcalcifications) and 12 mm (BIRADS 5) (Figure 4). The breast ultrasound confirmed three solid irregular hypoechoic nodules measuring 11 mm, 9 mm, and 12 mm, as well as some right axillary lymphadenopathy, the largest measured 25 mm (Figure 5). Histological examination of an ultrasound-guided biopsy revealed invasive carcinoma “no special type” (NST) according to the 2019 World Health Organization (WHO) classification, with a grade 2 histology (G2) with occasional outbreaks of ductal carcinoma and axillary lymph node metastasis. Additionally, a bone scan revealed no evidence of the disease’s metastatic localization (Figure 6). A multidisciplinary team decided to proceed with a right mastectomy and a right axillary dissection. The final histopathological report showed an invasive multifocal NST grade 2 carcinoma, positive for ER (98%), PR (80%), Ki67 (32%) and negative for HER2 (Luminal B HER2 negative). The resection margins were free of neoplastic infiltration. Two of the 14 lymph nodes were positive for invasive carcinoma. The final staging was G2 pTle pNla (2/14) MO, stage IIA. The patient received hormone therapy (Exemestane 25 mg daily and Decapeptyl 3.75 mg daily) due to high hormone receptor expression. Surgical treatment of the primary tumor did not improve neurological symptoms, prompting the patient to undergo intravenous steroid therapy; however, this also did not lead to any clinical improvement. The patient was evaluated at 2, 6, 12 and 24 months with clinical, radiological and serological examinations. On vestibular examination 2 months post-surgery, subjective dizziness and instability persisted. On video-oculoscopy, the patient presented ocular flutter with intermittent horizontal saccades especially in the lateral gaze. Total body CT scan (Figure 7) and breast ultrasound (Figure 8) at 2 months were negative for neoplastic recurrence or metastases. Serology for anti-Ri antibodies remained positive. Six months after surgery, the patient, who had been on Prednisone 50 mg/day, was admitted to the neurology department of another hospital. Patient reported an improvement in headache and a reduction in the frequency of emesis, but no improvement of the ocular and vestibular symptoms. The neurological examination documented oculovestibular syndrome with ocular flutter that was not inhibited by fixation, forcing the patient to keep her eyes closed, ataxia, head and trunk tremor, hypomimic facies, hypertonia in the right upper limb with difficulty in movements of the right hand, difficulty getting up from the chair without support, instability in open and closed eyed Romberg test, moon facies due to iatrogenic Cushing’s, slow, diprosodic and hypophonic speech. During admission, an MRI of the brain and brainstem was performed, which showed a slight enlargement of the peri-brain spaces in the frontal area and of the peri-brain spaces in the cerebellar area, possibly indicative of initial atrophy. Blood tests confirmed the presence of anti-Ri antibodies. Considering the immune-mediated genesis of the paraneoplastic syndrome, the patient underwent five sessions of plasmapheresis followed by a course of intravenous immunoglobulin (0.4 g/kg/day) with a slight and temporary clinical improvement. Due to the presence of a mild right rigid-kinetic hemi-syndromes with associated hypomimia and hypophonia, a genetic study for Parkinson’s disease was conducted and L-DOPA therapy was prescribed, which was then discontinued as the patient reported worsening symptoms. The patient underwent brain SPECT with receptor tracer to evaluate the presynaptic dopaminergic system the basal ganglia, which showed no impairment of the nigrostriatal presynaptic dopaminergic system. Due to the lack of response to chronic steroid therapy and the development of clinical signs of iatrogenic Cushing’s, following endocrinological consultation, the dosage of Prednisone was progressively reduced and then replaced by Cortone Acetate. Bone mineral density was normal. After one month, the patient was transferred to a rehabilitation center to undergo neuromotor and vestibular rehabilitation until the patient was transferred back to the prior neurology department due to a clinical worsening, in particular of the eye complaints. The patient was unable to perform more than a few steps without support, showed multidirectional fluctuations on the Romberg test with trunk instability. At eye opening there was persistence of the ocular flutter, with mydriatic pupils (left > right) and a cloudy but present response to direct light stimulus. The other cranial nerves were intact. Thermal tactile and pain sensitivity were preserved. Osteotendinous reflexes were brisk and symmetrical in the upper limbs and hypotensive in the lower limbs. Coordination tests (index-nose, index-index, finger-tapping), revealed mild motor impairment on the right side with kinetic and postural tremor and fine tremors in the upper limbs. During hospitalization, the patient underwent a new course of plasmapheresis (four sessions in 8 days) combined with intravenous immunoglobulins (0.4 g/kg/day for 4 days) with slight clinical benefit. Due to the need for chronic immunosuppressive treatment, oral therapy with Azathioprine at an initial dosage of 25 mg/day, later increased to 50 mg, was administered. A contrast-enhanced MRI of the brain and cervical spine was performed, which showed essentially unchanged findings compared to the previous check-up, including a modest enlargement of the peri-brain spaces in the frontal area and peri-foliar spaces in the cerebellum, expressing initial signs of atrophy. Slight clinical improvement was documented during hospitalization, particularly in head tremor and ocular flutter, allowing the patient to fixate, despite the persistence of pathological eye movements. Gait was improved, although the patient remained ataxic. In view of this progress, the patient was again transferred to the neuromotor rehabilitation center for one month at the 12 months follow-up visit, the patient reported a worsening of the oculovestibular syndrome with inexhaustible flutter preventing fixation, worsening of rigidity in the right upper limb and occasional dysphagia. The neurological examination showed dystonic flexion in the right upper limb and plastic hypertonia in the upper limbs bilaterally (right > left), but no hypertonia in the lower limbs. Independent ambulation was not possible. The patient had an intravenous infusion of cyclophosphamide, which brought only slight and transient improvements in symptoms. After 24 months from the surgery, the patient was re-evaluated in our hospital, and a vestibular examination via oculoscopy confirmed the presence of the flutter with horizontal saccades, which were slightly reduced compared to the first assessment in 2022. Vestibulospinal tests revealed a severe postural deficit, with ataxic walking and right upper limb rigidity.

## 3. Discussion

PNSs have an unclear pathogenesis. The most probable hypothesis is that antibodies or cytotoxic T-cells cause a cross-reaction between the cancer and certain central or peripheral neural tissues, which leads to a paraneoplastic condition [11,12]. The discovery of anti-neuronal or glial autoantibodies, which are helpful clinical biomarkers, supports the immune-mediated cause of PNSs. Onconeural antibodies differ from surface antibodies in both diagnosis (ELISA and immunoblotting search vs. immunohistochemistry) and treatment (favorable in most diseases associated with surface antibodies, but rather poor in tumor-associated cases with proven onconeural antibodies) [13]. Anti-Ri antibodies identify two proteins that bind RNA, Nova-1 and Nova-2, located in the nucleus of neuronal cells in the central nervous system. Brain MRIs and CS studies can be used to confirm the diagnosis of anti-Ri syndrome [14]. Specific autoantibodies can be associated with a peculiar clinical syndrome or a certain type of tumor. Anti-Ri antibodies are more commonly associated with subacute neurological paraneoplastic syndrome, which occurs more frequently with opsoclonus-myoclonus syndrome and paraneoplastic cerebellar degeneration with ataxia [15]. Atypical symptoms are jaw dystonia, laryngospasms, cranial nerve palsies, and other oculomotor disorders [16,17,18,19]. The current case is an unusual and rare presentation of anti-Ri syndrome with ocular flutter, subjective dizziness, instability, and photophobia. Anti-Ri syndrome has an immune-mediated pathogenesis supported by the frequent presence of neuronal specific antibodies [20]. So, the diagnosis is based on the physical examination that shows neurological symptoms, the research of a tumor through radiological investigations, and is supported by the search for onconeural antibodies in the serum. Both ocular flutter and opsoclonus are concerning ophthalmic findings due to their potential association with life-threatening conditions. They consist of bursts of consecutive involuntary conjugate binocular saccadic movements that distract the eyes from the primary position. In ocular flutter, the trajectory is strictly in the horizontal plane, and it may be in any plane. The exact etiology of ocular flutter and opsoclonus is unknown. It has been proposed that opsoclonus and flutter result from dysfunction of omnipause neurons (OPNs). The OPNs lie in the midbrain very close to the midline near the rostral pole of the abducens nucleus, in the intercalated raphe nucleus [21]. The treatment of opsoclonus and ocular flutter focuses on addressing the underlying cause; however, the ocular motility disorder often persists even after successful management of the tumor, as seen in our patient [22]. Thanks to a strong clinical suspicion of a paraneoplastic syndrome, a very early diagnosis of a small malignant lesion, which might not have been biopsied otherwise, was fortunately made. If tumor is not found, follow-up tests should be performed every 6 months for up to 4 years, except for Lambert–Eaton Miasthenic Syndrome (LEMS), where screening for 2 years is sufficient [23]. There is no standardized treatment for paraneoplastic syndrome (PNS). Early cancer treatment seems to improve neurological symptoms [17,19,23], but this did not occur for our patient. As already described in the literature, paraneoplastic syndromes with onconeural antibody positivity and vestibular system involvement are rarely reversible and difficult to treat. The therapies used typically allow only a modest and temporary reduction in symptoms. The only therapeutic strategy that appears to have been slightly effective in our specific case was high-dose intravenous immunoglobulin (IVIG) infusion combined with plasmapheresis and vestibular rehabilitation. Beyond the clinical and radiological findings, the patient’s functional status and quality of life outside of hospital settings were significantly impacted throughout the course of the disease. Despite periods of slight clinical improvement following immunomodulatory treatments, the patient remained unable to ambulate independently and required continuous assistance with daily activities. Caregiver reports during follow-up visits highlighted persistent fatigue, visual discomfort, and a high degree of frustration related to the loss of autonomy. Social participation and return to work were not possible due to the disabling oculovestibular symptoms, emotional burden, and motor limitations. These observations emphasize the need for a multidisciplinary approach that not only addresses the neurological aspects of the syndrome, but also supports the patient’s psychological well-being and daily functioning.

Rehabilitation plays a crucial role in improving the patient’s quality of life, even in the absence of complete symptom resolution [24]. In patients with anti-Ri syndrome presenting with ocular flutter and vestibular symptoms, rehabilitation aims to achieve the following objectives: improve balance and posture, reducing the risk of falls, enhance oculomotor stability to minimize visual disturbances and nausea, improve motor coordination and limb mobility, reduce spasticity and muscle rigidity through relaxation and stretching techniques, preserve residual function and prevent secondary disability. Rehabilitation therapy must be multidisciplinary, combining physical therapy, vestibular therapy, and neuromotor re-education. Vestibular rehabilitation is essential for managing balance disturbances and postural instability associated with ocular flutter and paraneoplastic cerebellar ataxia. Vestibular adaptation exercises include gaze stabilization (focusing on a static and moving target) to reduce oscillopsia and slow pursuit and saccadic eye exercises to improve ocular control. Balance re-education techniques include static and dynamic training with unstable surfaces and exercises in upright posture with progressively reduced visual support. Motor and postural rehabilitation are essential for managing ataxia and muscle weakness in anti-Ri syndrome. Key interventions include proprioceptive exercises and postural re-education to improve balance and coordination, treadmill therapy with weight support for gait re-education, and muscle relaxation techniques to reduce rigidity in the upper limbs. These therapies help enhance mobility, coordination, and overall functional recovery, improving patients’ independence and quality of life. In anti-Ri syndrome with ocular flutter, visual re-education is essential for improving eye movement control. It focuses on reducing saccadic hypermetria, enhancing fixation, training convergence to reduce visual discomfort, and integrating vestibular-ocular exercises for better spatial awareness. The expected outcomes include improved fixation, reduced diplopia, and enhanced visual stability, leading to a better quality of life for patients. Although rehabilitation cannot reverse the neuronal damage caused by the autoimmune response, it can still improve quality of life by reducing vestibular and motor symptoms, prevent secondary complications (muscle atrophy, tendon contractures) and promote the maintenance of autonomy for as long as possible. When personalized and integrated with immunosuppressive therapies, rehabilitation can be an essential pillar in the treatment of anti-Ri syndrome. While not curative, it allows for the optimization of residual functions and the improvement of the patient’s well-being [24].

The lack of neurological improvement suggests irreversible neuronal damage, despite the removal of the tumor. The prognosis of PNS is more influenced by the aggressiveness of the autoimmune response than by the underlying neoplasia.

Given the immune-mediated pathogenesis of the oculovestibular syndrome, a therapeutic approach utilizing biological drugs that interfere with the immune response may prove useful and effective. However, currently, few studies have been conducted on this matter.

In recent years, more targeted biological drugs and immunomodulatory strategies have been developed [25,26,27,28]. Several of these treatments have shown promising results in PNSs and associated autoimmune diseases [25,26]. Pro-inflammatory Cytokine Inhibitors, such as Tocilizumab (anti-IL-6R) [28], which targets IL-6, have shown favorable outcomes in autoimmune encephalitis and neuropathies associated with PNSs, particularly in corticosteroid-resistant cases, suggesting potential for reducing chronic neuroinflammation in anti-Ri PNSs. Additionally, Infliximab and Adalimumab, which block TNF-α, are already in use for systemic autoimmune diseases and are currently being trialed for PNSs, potentially limiting neuronal damage mediated by microglia and chronic inflammation.

Lymphocyte depletion therapies also show promise, as PNSs are mediated by cytotoxic T lymphocytes and antibodies. Rituximab, which depletes B lymphocytes and reduces the production of onconeural antibodies, has been successfully used in autoimmune encephalitis and Sjögren’s syndrome [28,29]. Ocrelizumab, a next-generation anti-CD20 therapy [30], offers improved tolerability and could be an option in cases where Rituximab fails. Another promising drug, Eculizumab, inhibits complement activation and has already been used in conditions like myasthenia gravis and neuromyelitis optica, with potential to protect neurons from complement-mediated destruction in PNSs [30].

Immune checkpoint therapies, such as Abatacept [29], which blocks T lymphocyte co-stimulation, and Belatacept, which prevents the proliferation of pathogenic T lymphocytes, offer potential to regulate the autoimmune response in PNSs. These therapies could prevent the activation of autoreactive T lymphocytes, thus reducing neuronal destruction in conditions like anti-Ri PNSs.

For microglia modulation, drugs like Ibudilast [31] and Minocycline [27] are being explored due to their ability to reduce microglial activation and the production of inflammatory cytokines. These agents may protect neurons from neuroinflammation without overly suppressing the immune system, offering potential in treating neurodegenerative diseases and paraneoplastic cerebellar degeneration.

Finally, gene and cell-based therapies represent the cutting edge of immunotherapy in PNS treatment. CAR-T cells, engineered to target autoreactive B lymphocytes [32], could provide a long-term solution for refractory PNSs, while Mesenchymal Stem Cell (MSC) therapies [33], known for their immunoregulatory and neuroprotective properties, offer promise for repairing neuronal damage caused by anti-Ri syndrome. Since anti-Ri syndrome is a PNS with irreversible neuronal damage, the priority is to block autoimmunity as early as possible. New immunomodulatory therapies are revolutionizing the treatment of PNSs resistant to traditional therapies. In anti-Ri syndrome, the most promising treatments include: Lymphocyte depletion (Rituximab, Ocrelizumab) to reduce antibody production, Cytokine inhibition (Tocilizumab, anti-TNF) to limit neuronal damage, Neuroprotection (Ibudilast, Minocycline) to prevent cerebellar atrophy, Cell-based therapies (CAR-T, stem cells) as future prospects. The future of PNS treatment may involve a combination of targeted immunotherapy and neuroprotection to enhance long-term outcomes.

## 4. Conclusions

In conclusion, the presented case underscores the importance of a thorough vestibular and clinical evaluation, which enabled the identification of saccadic anomalies such as ocular flutter. This symptom can be indicative of underlying malignancies, and therefore, clinicians should maintain a high index of suspicion. Early identification of such tumors can significantly enhance treatment outcomes, as many solid tumors may be amenable to surgical intervention if diagnosed early. An aggressive investigative approach, including appropriate imaging and laboratory tests, is crucial in establishing a timely diagnosis and facilitating effective treatment strategies. This proactive stance not only aids in improving patient prognosis but also emphasizes the essential role of ophthalmological signs in the broader context of systemic disease. There is no standardized treatment for paraneoplastic syndrome, and management of anti-Ri syndrome requires a personalized approach, combining vestibular rehabilitation and immunosuppressive therapies. Recent advancements in immunomodulatory treatments are transforming the management of PNSs that are resistant to traditional therapies.

## Figures and Tables

**Table 1:** Timeline of key clinical events.

Timepoint	Event Description
T—6 months	Onset of symptoms: subjective dizziness, oscillopsia, blurred vision, photophobia, severe vomiting → 16 kg weight loss over 7 months.
Month 0	Initial clinical evaluation: ocular flutter documented by video-oculoscopy. Normal brain MRI, CT scan, ophthalmological, serological, and metabolic workup. Anti-Ri antibodies positive (1:126). Whole-body CT, mammography, and breast ultrasound: suspicious right breast nodules and right axillary lymphadenopathy. Core needle biopsy: invasive NST breast carcinoma, G2, with nodal metastasis. Surgery: right mastectomy and axillary dissection. Initiation of hormonal therapy (Exemestane 25 mg daily + Decapeptyl 3.75 mg monthly) and Prednisone 50 mg/day.
Month 2	Clinical follow-up: persistent ocular flutter and vestibular symptoms. Anti-Ri antibodies still positive. Whole-body CT scan and breast ultrasound: no evidence of recurrence or metastases.
Month 6	Neurological hospitalization: worsening neurological symptoms. Brain MRI: early cerebellar atrophy. High-dose prednisone: limited effectiveness, iatrogenic Cushing’s syndrome. First plasmapheresis + IVIG (0.4 g/kg/day): slight, transient improvement. L-Dopa trial: discontinued due to worsening. Brain SPECT: normal dopaminergic system.
Months 7–8	Steroid taper and switch to cortisone acetate. First neuro-rehabilitation admission for neuromotor and vestibular therapy.
Months 9–11	Clinical deterioration → second neurological hospitalization. Persistent ocular flutter, truncal instability, ataxia, right upper limb rigidity. Second plasmapheresis (4 sessions) + IVIG: modest benefit. Azathioprine started (25 mg, increased to 50 mg/day). Re-admission to rehabilitation center.
Month 12	Follow-up: worsening oculovestibular syndrome, rigidity, and occasional dysphagia. Intravenous cyclophosphamide: minimal, temporary benefit.
Month 24	Two-year follow-up: ocular flutter still present (slightly reduced). Severe postural deficit, ataxic gait, right upper limb rigidity. No radiological evidence of tumor recurrence.

## Data Availability

The data presented in this study are available on request from the corresponding author due to privacy.

## Supplementary Materials

The following supporting information can be downloaded at: https://www.mdpi.com/article/10.3390/brainsci15060628/s1. Video S1: Video-oculography recording showing intermittent bursts of horizontal saccadic eye movements without intersaccadic intervals, consistent with ocular flutter. The movements are conjugate and limited to the horizontal plane.
