# Supplementary material for: Anti-Ri Paraneoplastic Neurological Syndrome Presenting with Ocular Flutter in a Patient with Breast Cancer
Source: Brain Sci. 2025 Jun 11;15(6):628. doi: 10.3390/brainsci15060628 (PMC12191030; doi:10.3390/brainsci15060628)
Supplement: Supplementary file 1 [file brainsci-15-00628-s001.zip › brainsci-3606220-supplementary.docx]

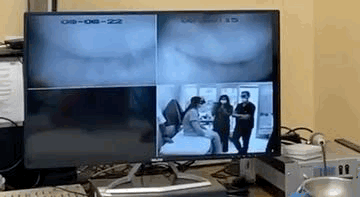


**Supplementary Video S1.** Video-oculography recording showing intermittent bursts of horizontal saccadic eye movements without intersaccadic intervals, consistent with ocular flutter. The movements are conjugate and limited to the horizontal plane.
